# The relationship between depressive symptoms among female workers and job stress and sleep quality

**DOI:** 10.1186/2052-4374-25-12

**Published:** 2013-07-22

**Authors:** Ho-Sung Cho, Young-Wook Kim, Hyoung-Wook Park, Kang-Ho Lee, Baek-Geun Jeong, Yune-Sik Kang, Ki-Soo Park

**Affiliations:** 1 Department of Occupational & Environmental Medicine, Samsung Changwon Hospital, School of Medicine, Sungkyunkwan University, MD, Korea; 2 Department of Preventive Medicine, School of Medicine, Gyeongsang National University, MD, Korea

## Abstract

**Objective:**

Recently, workers' mental health has become important focus in the field of occupational health management. Depression is a psychiatric illness with a high prevalence. The association between job stress and depressive symptoms has been demonstrated in many studies. Recently, studies about the association between sleep quality and depressive symptoms have been reported, but there has been no large-scaled study in Korean female workers. Therefore, this study was designed to investigate the relationship between job stress and sleep quality, and depressive symptoms in female workers.

**Methods:**

From Mar 2011 to Aug 2011, 4,833 female workers in the manufacturing, finance, and service fields at 16 workplaces in Yeungnam province participated in this study, conducted in combination with a worksite-based health checkup initiated by the National Health Insurance Service (NHIS). In this study, a questionnaire survey was carried out using the Korean Occupational Stress Scale-Short Form(KOSS-SF), Pittsburgh Sleep Quality Index(PSQI) and Center for Epidemiological Studies-Depression Scale(CES-D). The collected data was entered in the system and analyzed using the PASW (version 18.0) program. A correlation analysis, cross analysis, multivariate logistic regression analysis, and hierarchical multiple regression analysis were conducted.

**Results:**

Among the 4,883 subjects, 978 subjects (20.0%) were in the depression group. Job stress(OR=3.58, 95% CI=3.06-4.21) and sleep quality(OR=3.81, 95% CI=3.18-4.56) were strongly associated with depressive symptoms. Hierarchical multiple regression analysis revealed that job stress displayed explanatory powers of 15.6% on depression while sleep quality displayed explanatory powers of 16.2%, showing that job stress and sleep quality had a closer relationship with depressive symptoms, compared to the other factors. The multivariate logistic regression analysis yielded odds ratios between the 7 subscales of job stress and depressive symptoms in the range of 1.30-2.72 and the odds ratio for the lack of reward was the highest(OR=2.72, 95% CI=2.32-3.19). In the partial correlation analysis between each of the 7 subscales of sleep quality (PSQI) and depressive symptoms, the correlation coefficient of subjective sleep quality and daytime dysfunction were 0.352 and 0.362, respectively.

**Conclusion:**

This study showed that the depressive symptoms of female workers are closely related to their job stress and sleep quality. In particular, the lack of reward and subjective sleep factors are the greatest contributors to depression. In the future, a large-scale study should be performed to augment the current study and to reflect all age groups in a balanced manner. The findings on job stress, sleep, and depression can be utilized as source data to establish standards for mental health management of the ever increasing numbers of female members of the workplace.

## Introduction

Since the currency crisis in 1997, Korea has experienced dramatic social and economic changes. Large and small businesses went bankrupt or underwent layoffs, staff reduction and restructuring. With the gradual deterioration of the work environment, many workers have been exposed to various kinds of job stress, including job insecurity [1,2]. Under these circumstances, workers' mental health has recently drawn a great deal of attention in the field dealing with occupational health management.

According to 2010 data of the Korean National Statistical Office [3], there were 15,413 suicides in 2009, an increase of 2,555 (19.9%) compared with the previous year. It also reported that 31.0 out of every 100,000 people committed suicide, an increase of 19.3% from the previous year, leading to the highest suicide rate among the member nations of the Organization for Economic Cooperation and Development (OECD) [4]. People with experience of a suicide attempt were shown to be significantly associated with mental illness, especially depression, which is one of the most prevalent psychiatric illnesses and which may cause serious problems like suicide if it remains untreated [5,6]. An investigation of Korean mental health in 2006 showed that the lifetime prevalence of depression was 5.6%, increased from 4.0% in 2001 [7]. In addition to its influence on individual mental health, depression results in a considerable socio-economic burden [8], leading to reduced productivity in the workplace. Thus, there is an urgent need to pay attention to the depressive symptoms of workers from a social perspective [9-11].

The relationship between job stress, among many factors that may impact depression, and depressive symptoms has already been demonstrated in many studies [12-15]. It has also been reported that social psychological working conditions such as lack of reward, interpersonal conflict, and poor social support may serve as stress factors and adversely affect depression [16]. In addition, recently, many studies have been conducted about the relationship between sleep and depressive symptoms, including a study of Lee et al [17]. Investigating the relationship between sleep quality and depressive symptomsand Nakata's study [18] reporting that shorter sleeping time and poorer subjective sleep quality is associated with higher a risk of depressive symptoms.

According to previous Korean and international studies [19,20], women have a 2- to 3- times higher chance of having depression over the course of a lifetime than do men. Moreover, despite the fact that more and more women are working outside the home and have their own working life, they still suffer from a higher burden surrouding childbirth and child-raising duties and also have higher job stress than men due to unfair treatment in hiring, promotion, remuneration, and task assignments.

Although female workers are relatively more susceptible to depression and job stress factors, previous Korean studies related to depression have been conducted mostly in male workers with limited consideration of females, focusing on major companies and small and medium-sized companies [12,13,21] or special occupational categories such as office workers in automobile companies [22] and service workers [14,23]. To fill the gap, we aimed to study the relationship between job stress and sleep quality, on the one hand, and depressive symptoms, on the other, in female workers in manufacturing, finance, and service fields.

## Materials and methods

### Subjects

From Mar 2011 to Aug 2011, self-administered questionnaires were collected from 4,966 female workers in manufacturing, finance, and service fields at 16 0077orkplaces in Yeungnam province while worksite-based health checkups were conducted by the National Health Insurance Service (NHIS). Among them, 4,833 female workers were included in the final analysis, excluding 83 with incomplete responses. This study was reviewed by the institutional review board (IRB) before implementation and all questionnaires were obtained under consent of the participants(IRB No. 2012-SCMC-059-00).

### Study variables and measurements

#### General characteristics

Socio-demographic characteristics and health behaviors such as age, educational level, marital status, body mass index (BMI), smoking habit, alcohol drinking, regular exercise, and job tenure (years) were reviewed. The subjects' age ranged from 17 to 59 years. They were categorized by age (≤24 years; 25–29 years; or ≥30 years), educational level (high school diploma or below; or college entrance or higher) and marital status (unmarried; married; divorced; or divorced or widowed). For the obesity level, WHO criteria pertaining to obesity for Asia-Pacific region was employed: BMI < 18.5 kg/m^2^ indicating low weight; BMI ≥18.5 and ≤ 24.9 kg/m^2^ indicating normal weight; and BMI ≥25 kg/m^2^ indicating obesity [24]. Among the factors of health behavior, smoking habit was categorized into non-smoker; ex-smoker; and current smoker status, while alcohol drinking was classified based on the mean alcohol consumption per week. Regarding regular exercise, subjects were classified into thoese who did or did not exercise at least 3 times a week, regardless of the intensity. Lastly, job tenure was categorized into the following groups: < 1 year; 1–4 years; 5–9 years; and ≥10 years.

#### Job stress

The Korean Occupational Stress Scale-Short Form(KOSS-SF) [25] was used as a tool to determine job stress. The KOSS-SF consists of 24 questions under 7 subscales: job demand, insufficient job control, interpersonal conflict, job insecurity, organizational system, lack of reward and occupational climate. Each question uses a 4-point Likert scale(1-2-3-4). A higher score indicates relatively higher job stress. Questions whose higher score indicates low job stress were reciprocally re-encoded. The Total KOSS-SF scores and 7 subscale scores were converted into a 100-point system. The Total job stress score and subscale scores were dichotomized based on the median of the converted scores in the KOSS-SF into a high stress group (top 50%) and low stress group (low 50%) for analysis of the relationship with depressive symptoms.

#### Sleep quality

Sleep quality was assessed using the Korean language version of the Pittsburgh Sleep Quality Index(PSQI) [26], originally developed by the University of Pittsburgh and translated by Kim [27]. The PSQI consists of 7 items including subjective sleep quality, sleep latency, sleep duration, habitual sleep efficiency, sleep disturbances, use of sleeping medication, and daytime dysfunction. The Total possible score is 21 points, and a higher score indicates poorer quality of sleep. This study employed a 5-point threshold indicating a sleep disorder suggested by Buysee et al. [26] people with 5 or more points were categorized into a poor sleep quality group, while people below 5 points were good sleep quality group.

#### Assessment of depressive symptoms

The depression level was assessed using a validated Korean translation by Cho et al. [28] of the self-reported Center for Epidemiological Studies-Depression Scale(CES-D) developed by National Institute of Mental Health(NIMH) in 1971 [29]. This questionnaire consists of 20 questions. Each question uses a 4-point Likert scale (0-1-2-3) and higher scores indicate higher depression. Questions whose higher score indicates low depression were reciprocally re-encoded. The total possibe score is 60 points. Based on the threshold of 21 points to screen Korean community-dwellers with depressive symptoms [28], subjects with a score 21 or more points were classified as people with depressive symptoms.

### Analysis methods

Socio-demographic characteristics, smoking habit, lifestyle, job characteristics, job stress and sleep quality with subscales were summarized using descriptive statistics with frequencies and percentages. The t-test and ANOVA test were employed to analyze the level of depressive symptoms using each factor as an independent variable. To determine the prevalence, the chi-squared test was used based on the total CES-D score of 21. A multivariate logistic regression analysis was conducted to determine the impact of job stress, the 7 subscales, and sleep quality on depressive symptoms and the significance.

To investigate the stepwise influence of independent variables on depressive symptoms, hierarchical multiple regression was performed using a variable with significant results in univariate analysis as an independent variable and the depression level score as a dependent variable. All Variation Inflation Factor(VIF) values to confirm multi-collinearity between variables were below 2 points, not showing any specific issues regarding multi-collinearity. When a significant independent variable was not a continuous variable, it was converted to dummy variable for use. Logistic regression analysis was conducted among the 7 subscales of job stress and depressive symptoms, while multiple regression analysis was conducted among the 7 subscales of sleep quality and depressive symptoms. The collected data was entered in a system and statistically analyzed using the PASW (version 18.0) program at a statistical significance of 0.05.

## Results

### Distribution of the depression group according to general characteristics

Based on the 21-point threshold of the total CES-D score, 978 subjects (20% of all subjects) obtained 21 or more points (Table 1). In the distribution by age, the majority were in their 20s, accounting for 89.5% of all of the subjects: 2,396 subjects (49.1%) ≤24 years and 1,971 subjects (40.4%) 25–29 years of age. Among the subjects, 516(10.6%) were ≥ 30 years. The study results showed that those in their 20s tended to have more depression: 24.9% ≤24 years old and 17.5% in the 25- to 29- year-old group, which was significantly higher than 7.2% in the group ≥ 30 years of age (p<0.001). The proportion with depression was also significantly higher in the low education group (24.2%) versus the high education group (14.1%); and in the unmarried (22.3%) and divorced or widowed (33.1%) versus the married group (9.5%) (p<0.001). The low weight group (20.9%) and obese group (26.1%) showed higher depression than the normal weight group. The depression rate in the alcohol drinking group ≥ 1 times per week (21.6%) was significantly higher than that in the no drinking group (< 1 times per week) (17.9%). As for job characteristics, the job tenure data showed a statistically significant difference in the depression rate of the 1–4 year group (24.5%) (p<0.001). However, the distribution of depression according to smoking habit and regular exercise did not show statistically significant differences.

**Table 1:** Distribution of depression according to general characteristics

**Variable**	**Number (%)**	**Mean**	**CES-D score N (%)**		*p*-value^*^
			**<21**	**≥21**	
Age (years)					<0.001
≤24	2,396(49.1)	14.6	1,799(75.1)	597(24.9)	
25-29	1,971(40.4)	12.5	1,627(82.5)	344(17.5)	
≥30	516(10.6)	9.4	479(92.8)	37(7.2)	
Educational level					<0.001
≤High school	2,852(58.4)	14.6	2,161(75.8)	691(24.2)	
≥College	2,031(41.6)	11.2	1,744(85.9)	287(14.1)	
Marital status					<0.001
Unmarried	3,967(81.2)	14.0	3,082(77.7)	885(22.3)	
Married	887(18.2)	9.7	803(90.5)	84(9.5)	
Divorced or widowed	29(0.6)	16.9	20(69.0)	9(31.0)	
BMI (kg/m^2^)					<0.001
<18.5	727(14.9)	13.1	575(79.1)	152(20.9)	
18.5-24.9	3,602(73.8)	12.9	2,925(81.2)	677(18.8)	
≥25.0	554(11.3)	15.0	405(73.1)	149(26.9)	
Smoking habit					0.993
Non-smoker	2,888(59.1)	13.3	2,311(80.0)	577(20.0)	
Ex-smoker	699(14.3)	13.1	558(79.8)	141(20.2)	
Current smoker	1,296(26.5)	13.0	1,036(79.9)	260(20.1)	
Alcohol drinking					<0.001
<1 time per week	2,081(42.6)	12.7	1,709(82.1)	372(17.9)	
≥1 time per week	2,802(57.4)	13.6	2,196(78.4)	606(21.6)	
Regular exercise					0.755
<3 times per week	1,279(26.2)	13.3	1,019(79.7)	260(20.3)	
≥3 times per week	3,604(73.8)	13.2	2,886(80.1)	718(19.9)	
Job tenure (years)					<0.001
<1	179(3.7)	11.2	153(85.5)	26(14.5)	
1-4	1,621(33.2)	14.4	1,224(75.5)	397(24.5)	
5-9	2,540(52.0)	13.1	2,050(80.7)	490(19.3)	
≥10	543(11.1)	10.7	478(88.0)	65(12.0)	
Total	4,883(100.0)	13.2	3,905(80.0)	978(20.0)	<0.001

^*^comparison by chi-squared test.

### Associations between job stress, sleep quality and depressive symptoms

In the KOSS-SF, the odds ratio signifying that the high job stress group was more likely to belong to the depression group than the low job stress group was 3.72(95% CI=3.18-4.35) (Table 2). In addition, when the subjects were divided into the good sleep quality group and poor sleep quality group based on a 5-point threshold of the PSQI, the odds ratio signifying that the poor group was more likely to belong to the depression group compared to the good group was 4.30(95% CI=3.60-5.14). Variables showing a significant impact on depression conditions in univariate analysis - age, marital status, educational level, BMI, alcohol drinking, and job tenure - were adjusted for multivariate logistic regression analysis. The results showed that the odds ratio of the high job stress group developing of depression was 3.58(95% CI=3.06-4.21) and that of the poor sleep quality group developing depression was 3.81(95% CI=3.18-4.56).

**Table 2:** Associations between job stress and sleep quality, and depressive symptoms

**Variables**	**Number (%)**	**Crude OR**			Adjusted OR*		
		**OR**^†^	**95% CI**^‡^		**OR**^†^	**95% CI**^‡^	
KOSS-SF^§^							
Low	2,464(50.5)	1.00			1.00		
High	2,419(49.5)	3.72	3.18	4.35	3.58	3.06	4.21
PSQI^∥^							
Good	2,001(41.0)	1.00			1.00		
Poor	2,882(59.0)	4.30	3.60	5.14	3.81	3.18	4.56

*adjusted by age, marital status, education level, alcohol drinking, BMI, job tenure.

^†^odds ratio, ^‡^confidence interval.

^§^Korean Occupational Stress Scale-Short Form, ^∥^Pittsburgh Sleep Quality Index.

### Hierarchical multiple regression of explanatory variables with depressive symptoms

Through hierarchical multiple regression analysis, socio-demographic characteristics other than smoking and regular exercise that were not significant in uivariate analysis, health behavior, and job tenure were entered into Model I as independent variables (Table 3). Age, marital status, educational level, and BMI were strongly associated with depressive symptoms: younger age, unmarried status, low educational level, and abnormal weight (low weight and obese) were factors for depression, showing a significantly high depression rate compared to each counterpart. Model I explained 6.9% of depressive symptoms. Model II included an additional independent variable, job stress (KOSS-SF), along with those in Model, showing that higher job stress is associated with a higher depression level. The additional variable of job stress increased the explanatory power to 15.6% in Model II. Model III employed sleep quality (PSQI) as an independent variable that may affect the depression level, showing that poor sleep quality significantly increased the depression level. The sleep quality variable added to Model III increased the explanatory power to 16.2%. Lastly, Model IV included the job stress (KOSS-SF) factor and sleep quality (PSQI) factor as independent variables for hierarchical regression analysis, showing that the additional two factors in Model IV increased the explanatory power to 26.1%.

**Table 3:** Hierarchical multiple regression of explanatory variables for depressive symptoms

**Variables**	**Model I**		**Model II**		**Model III**		**Model IV**	
	**B**	**t**	**B**	**t**	**B**	**t**	**B**	**t**
Age (year)	−0.223	−6.294*	−0.223	−6.891*	−0.182	−5.666*	−0.189	−6.299*
Marital status^†^								
Unmarried	2.965	7.148*	2.484	6.554*	1.493	3.929*	1.351	3.811*
Divorced/Widowed	8.181	4.431*	8.733	5.182*	7.451	4.439*	8.029	5.126*
Education level^†^	−2.931	−10.195*	−2.134	−8.092*	−2.030	−7.723*	−1.532	−6.227*
Alcohol drinking^†^	0.469	1.669*	0.643	2.507*	−0.044	−0.172*	0.187	0.782*
BMI (kg/m^2^)	0.196	4.389*	0.171	4.189*	0.091	2.243*	0.089	2.333*
Job tenure (years)	0.004	0.088*	0.042	1.068*	−0.037	−0.948*	0.001	0.033*
Job stress (KOSS-SF)			0.368	31.272*			0.301	26.934*
Sleep Quality (PSQI)					1.491	32.015*	1.235	27.756*
Constant	13.102		−4.061		7.592		−5.524	
F	51.557*		176.397*		182.709*		267.151*	
Adjusted R^2^	0.068*		0.223*		0.229*		0.329*	
R^2^ change	0.069*		0.156*		0.162*		0.261*	

Model I: adjusted by age, marital status, education level, alcohol drinking, BMI ,job tenure.

Model II: adjusted for modelI plus job stress (KOSS-SF).

ModelIII: adjusted for modelI plus sleep quality (PSQI).

ModelIV: adjusted for modelI plus job stress (KOSS-SF) and sleep quality (PSQI).

*p<0.001

^†^m0061rital status (0:married, 1:unmarried, 2:divorced/widowed).

education level (0:high school graduate, 1:college graduate).

alcohol drinking(0:no, 1:yes).

### Association between subscales of job stress and sleep quality and depressive symptoms

A logistic regression analysis showed the depression distribution according to job stress subscales where the high job stress group in all subscales had a statistically higher risk of depression compared to the low stress group (Table 4). The odds ratio was 3.09(95% CI=2.64-3.60) for lack of reward, 2.47(95% CI=2.14-2.86) for interpersonal conflict, 2.46(95% CI=2.13-2.84) for job demand, 2.41(95% CI=2.07-2.80) for occupational climate, 2.18(95% CI=1.89-2.51) for job insecurity, 2.14(95% CI=1.85-2.48) for organizational system and 1.48(95% CI=1.28-1.70) for insufficient job control.

**Table 4:** Associations between job stress subscales and depressive symptoms

**Subscales**	**Classification**	**Crude OR**		Adjusted OR*	
		**OR**^**†**^	**95% CI**^**‡**^	**OR**^**†**^	**95% CI**^**‡**^
Job demand					
	Low	1.00		1.00	
	High	2.46	2.13-2.84	2.10	1.81-2.45
Insufficient job control					
	Low	1.00		1.00	
	High	1.48	1.28-1.70	1.30	1.12-1.51
Interpersonal conflict					
	Low	1.00		1.00	
	High	2.47	2.14-2.86	2.32	2.00-2.70
Job insecurity					
	Low	1.00		1.00	
	High	2.18	1.89-2.51	2.24	1.93-2.60
Organizational system					
	Low	1.00		1.00	
	High	2.14	1.85-2.48	1.97	1.70-2.29
Lack of reward					
	Low	1.00		1.00	
	High	3.09	2.64-3.60	2.72	2.32-3.19
Occupational climate					
	Low	1.00		1.00	
	High	2.41	2.07-2.80	2.38	2.03-2.78

*adjusted by age, marital status, education level, alcohol drinking, BMI, job tenure, PSQI

^†^odds ratio, ^‡^confidence interval

When variables showing significant relevance to depressive symptoms in uivariate analysis such as age, marital status, educational level, BMI, alcohol drinking, job tenure, and sleep quality (PSQI) were adjusted, the odds ratio of the high stress group compared to the low stress group in each job stress subscale for depression was 2.72(95% CI=2.32-3.19) for lack of reward, 2.38(95% CI=2.03-2.78) for occupational climate, 2.32(95% CI=2.00-2.70) for interpersonal conflict, 2.24(95% CI=1.93-2.60) for job insecurity, 2.10(95% CI=1.81-2.45) for job demand, 1.97(95% CI=1.70-2.29) for organizational system and 1.30(95% CI=1.12-1.51) for insufficient job control. Lack of reward showed the highest odds ratio, while insufficient job control had the lowest odds ratio among the subscales.

In multiple regression analysis among the 7 sleep quality (PSQI) subscales and depressive symptoms (CES-D), regression coefficients of daytime dysfunction and subjective sleep quality were 3.05 and 2.32, respectively, showing higher values than the other 5 sleep quality subscales (Table 5): 1.26 for sleep latency, 0.44 for sleep duration, 0.70 for habitual sleep efficiency, 2.27 for sleep disturbance and 1.66 for use of sleeping medication.

**Table 5:** Multiple linear regression analyses of sleep quality subscales for depressive symptoms

**Subscales**	**β**	SE*	**t**	***p*-value**
Subjective sleep quality	2.32	0.226	10.256	<0.001
Sleep latency	1.26	0.156	8.074	<0.001
Sleep duration	0.44	0.152	2.868	0.004
Habitual sleep efficiency	0.70	0.193	3.592	<0.001
Sleep disturbances	2.27	0.294	7.716	<0.001
Use of sleeping medication	1.66	0.564	4.712	<0.001
Daytime dysfunction	3.05	0.165	18.524	<0.001

*standard error.

## Discussion

This study was designed to investigate the relationship among general characteristics, job stress, sleep quality, and depressive symptoms in 4,883 female workers in manufacturing, finance, and service fields. Female workers in the finance and service fields were employees of restaurants and financial institutions belonging to 16 manufacturing workplaces, accounting for less than 4% of all of the participants. Thus, the female workers involved in this study were mostly working in manufacturing and their depression rate was 20.0%. In a depression study conducted among general employees, Choi et al. [30]. has reported a depression rate of 15.5%, while in a general Korean population depression study of Park et al. [31] , the depression rate was 18.1%. Also Kim et al. [32] have also reported a depression rate of 19.2% in a study involving 17,457 firefighters. Compared to these previous studies, this study showed a higher depression rate. This can be explained by the higher percentage of young sensitive females in their 20s (89.5%) who are likely to have higher physical and emotional stresses arising from their first experience of organizational and structural occupation systems with hierarchical relationships and interpersonal conflict due to their low rank in the workplace. A study based on US National Comorbidity Survey(NCS) data [33] has reported a higher depression prevalence in young adults and a study of Park et al. [13] involving employees in small and medium-sized Korean companiesalso showed the highest depression rate in young female workers in their 20s compared to other age groups.

In association with general characteristics, the depression level was higher in the younger age group; unmarried, divorced or widowed group; lower education group; low weight or obese group; and alcohol drinking group. Factors that may contribute to a higher depression rate of female workers in their 20s compared to older female workers include job adjustment issue due to their relatively limited work experience and job stress caused by their low position. Unmarried and divorced or widowed groups who do not have support from family have a higher risk of depression. In addition, a low educational level was another contributor to depression, which was supported by a study involving depression patients in Turkey [34] that demonstrated that a low educational level was a risk factor of depression development, especially in females. Regarding the relationship between obesity and depression, the study showed similar results to previous studies [35,36] , demonstrating that young female workers have a greater interest in their appearance and thus receive significant stress from that and that obesity is correlated with low self-respect and passive interpersonal relations.

The depression rate according to job stress (KOSS-SF) was significantly higher in the high stress group than the low stress group. As a result of multivariate logistic regression analysis, the high stress group had a higher odds ratio for depression. This result is consistent with previous studies reporting that job stress is a cause of increased risk of mental health including depression [17,37,38].

The depression rate according to sleep quality (PSQI) was significantly higher in the poor sleep quality group than the good sleep quality group. As a result of multivariate logistic regression analysis conducted to determine the association between sleep quality and depressive symptoms, the poor sleep quality group showed a higher odds ratio than the good sleep quality group. This result was consistent with a study involving employees of small-scale manufacturing companies [17] that has reported the odd ratio of 3.90(95% CI=2.83-5.36).

As in studies suggesting that people with depression have a higher chance of insomnia [39,40], sleep quality is often interpreted as an endpoint of depression. However, Lee et al [41]. has reported that poor sleep increases fatigue, aggression, anxiety, and depression and interferes with social activities in the daytime. Like several previous studies, this study interpreted depressive symptoms as endpoints of sleep quality, paying attention to the impact of poor sleep quality on depressive symptoms [18,42].

In the hierarchical multiple regression analysis using 4 different models, the overall explanatory power of the variables associated with depressive symptoms was 32.9%. Among the factors that may affect depressive symptoms, the general characteristics employed in Model I showed a limited explanatory power of only 6.9%. Model II and Model III showed that both job stress and sleep quality have a substantial influence on depressive symptoms, while Model IV demonstrated a dramatic increase in the explanatory power of job stress and sleep quality together to 26.1%. This suggested that these two factors have a considerable synergic effect on depressive symptoms.

This study in female workers showed that job stress is a significant contributor to depressive symptoms. With the finding, a logistic regression analysis was conducted to confirm which job stress items of the 7 subscales can affect the depression level. It was found that all 7 subscales were strongly associated with depressive symptoms, among which the lack of reward showed the closest relationship, while insufficient job control showed the least. This was not consistent with a previous study of Kim et al [32]. that reported job insecurity to be the greatest factor. We assumed that the reason that lack of reward was the greatest factor in job stress associated with depression level in female workers was because staff reductions and remuneration can affect depression, as demonstrated in previous studies [43-45], and because the majority of participants in this study were young manufacturing workers in their 20s who earn relatively low wages. As reported by Larisch et al [46]. in a study involving German workers, a greater gap between the labor required and remuneration led to a stronger relationship with depressive symptoms, Thus the female workers in their 20s could have felt that they were not being paid as much as they deserved for their work. According to 2011 data from the Korean National Statistics Office [47], a significant gender wage gap remains in Korea: women's average earnings reached only 66.9% of men's in 2010. This reality could be a cause of women's job stress.

It has been found that among employees of small and medium-sized companies and office workers, the occupational climate was a significant factor associated with depressive symptoms [13,48]. Similarly, this study showed that female workers would also be expected to feel substantial job stress from unreasonable communication and a hierarchical environment in a male-centered workplace. Regarding interpersonal conflict, this study found different results from a previous study involving employees of small and medium-sized manufacturing companies [13] where interpersonal conflict was a significant factor for males associated with depressive symptoms, but not for females.

Multiple regression analysis was conducted to confirm the association between 7 subscales of sleep quality (PSQI) and depressive symptoms (CES-D) showed higher regression coefficients in subjective sleep quality and daytime dysfunction, compared to the other 5 subscales. This result can be interpreted to mean that rather than objective factors like sleep duration and use of sleeping medication, subjective factors assessed by the subjects themselves according to their own interpretation such as subjective sleep quality and daytime dysfunction indicating limited social activities and difficulty in work concentration due to sleepiness are closely related with depressive symptoms [18]. Therefore, we assumed that depressive symptoms associated with sleep quality are greatly influenced by subjective factors like satisfaction with sleep or sound sleep, than by the absolute duration of sleep.

The study had some limitations. First, given its cross-sectional study design, this study can determine the association among job stress, sleep quality, and depressive symptoms but may not be able to fully explain the causal relationship among them. Second, regarding sleep, the shift rotation factor was not reflected although it is closely related to sleep quality or sleep duration. In addition, because the proportion of young female workers in their 20s was remarkably higher than that of older female workers in their 30s and above, the results cannot be generalized as an assessment on the mental health of female workers across all age groups.

In spite of these limitations, this study is meaningful in that it has shown that the depressive symptoms of female workers are closely related to job stress and sleep quality, given the fact that females are relatively vulnerable to depression compared to males and the current situation in which more and more women are entering the workforce. A study with a large-scale sample should be performed in the future to supplement the study limitations and to identify occupational factors of the development of depression, reflecting all age groups. Based on this study, depression management standards for female workers should be established and a suicide prevention program should be developed and introduced. In addition, this study can be used as source data for mental health management in increasing female workforce across all fields.

## Competing interests

The authors declare that they have no competing interests.

## Authors’ contributions

YWK conceived and designed this study. HSC and YWK and BGJ were involved in writing the manuscript. HSC and KHL performed the data collection. HOP and KSP performed the statistical analysis, the interpretation of data. YSK had critically revised the manuscript. All authors read and approved the final manuscript.
